# A Surgical Tuberculosis Scheme

**Published:** 1920-09-11

**Authors:** 


### A Surgical Tuberculosis Scheme.

As is well known, the Ministry of Health is now urging sanitary authorities to make provision for the needs of their respective areas as regards surgical tubercle; and at a recent meeting of the Stoke-on-Trent Council the local Health Committee presented a scheme for a new hospital to be devoted to treatment of this disease. It appears that a communication was received from the Ministry of Health giving a time limit until June 30, 1920, failing production of a scheme by which date the grant of £180 per bed for the provision of institutional accommodation would not be available. Accordingly the scheme put forward depends for financial support upon the Government, the Corporation, and the Cripples' Aid Society, joint management to devolve upon the two last bodies. A hospital is suggested of 150 beds for cases of surgical tuberculosis amongst the poorer classes—the Corporation to provide 100 beds for tuberculosis and twenty-five beds for cripples up to five years of age, the Cripples' Aid Society twenty-five beds for other cases. An existing institution is proposed as an out-patient and after-care department by the latter body, and to be managed by it, a daily or weekly charge per patient being made. The scheme is to be managed by a surgical tuberculosis officer to the Corporation, who will act as resident medical officer of the institution, aided by a resident assistant and by a consulting staff of one physician, one surgeon, one laryngologist, and one anæsthetist. As to finance, the capital charges are estimated at £500 per bed, less, of course, the Government grant of £180. Half the loan charges (put down at 8 per cent.) would be recouped by the Ministry of Health, while for annual repayment and maintenance £100 per bed is allowed; again, half this annual expense is contributed by the State. With mention of a small additional outlay on the out-patient department, the description of the scheme, which the Council was recommended to adopt, closes.

We do not know, of course, the exact local conditions, and, perhaps, disabilities; but to begin with the last, and certainly not the least, item, namely, that of finance, it should be possible, even in these days of *la vie chère*, to get under a capital expenditure of £500 per bed. For the institutional accommodation of cases of surgical tubercle, probably the cheapest and best plan is that adopted at the lately opened branch of the well-known Treloar Hospital at Alton. Here the staff are accommodated in a largish house, not specially built, close to which has been erected a long narrow fifty-bed pavilion. It must be remembered that for many months of the year the patients need to be wheeled outside the wards all day, in order to be exposed to sunlight, while in the case of children, accommodation on upper floors is always associated with a certain amount of risk to life and limb. The wards must therefore have a long south front provided with a great number of doors, and be very shallow from back to front to make as little wheeling to and fro as possible. In short, they need be of very slight structure. The only substantial accommodation required is for the rooms for plaster work, for *x*-ray, and for the operating-theatre—which can very well be arranged for in the same house that shelters the staff. These specially built pavilions are so economical in the first place, and they enable attendance, and therefore the numbers of the staff, to be economised so greatly, that their use might almost be made obligatory in these days of building difficulties—here, as ever, we come on the need in the anti-tuberculosis campaign of a centralised control.

As to the description of patient catered for, we think it a mistake to mix up non-tuberculous children with tuberculous ones; a mistake on the grounds of possible infection, and of divergent requirements. Months and years of recumbency, heliotherapy, open air day and night, are not required for club foot, and it is waste therefore to provide them. Again, a consulting staff is not necessary for the conservative treatment of surgical tubercle, nor a special anæsthetist, if there are two residents. In fact, those familiar with the history of the gradual acclimatisation of these Continental methods in this country can tell of distinct obscurantism and obstruction proceeding from such a body. With these points kept in mind, the treatment of surgical tubercle, now at length being developed, should progress well. The much more important matter of prophylaxis is almost solely an economic problem; one might indeed say a political one.

